# AI‐Powered, Temperature‐Resolved Centrifugal Microfluidics for Rapid 3D Phase‐Diagram Generation of Biomolecular Condensates

**DOI:** 10.1002/advs.77523

**Published:** 2026-08-31

**Authors:** Jiashuo Li, Pengjie Li, Kaiting Zhang, Yongqi Guo, Peng Chen, Bi‐Feng Liu, Yiwei Li

**Affiliations:** ^1^ Key Laboratory of Molecular Biophysics of MOE and Hubei Bioinformatics and Molecular Imaging Key Laboratory Department of Biomedical Engineering College of Life Science and Technology Huazhong University of Science and Technology Wuhan China

**Keywords:** 3D phase diagram, biomolecular phase separation, centrifugal microfluidics, deep learning

## Abstract

Biomolecular phase separation drives the formation of membraneless organelles and is implicated in numerous diseases. Mapping multidimensional phase diagrams is essential for understanding condensate assembly mechanisms and developing therapeutic interventions, yet current methods are slow, resource‐intensive, and often limited to a single, frequently non‐physiological, temperature. Here, we present T‐PhaseMap, an AI‐powered centrifugal microfluidic platform that integrates programmable concentration‐gradient generation, standalone temperature control, and deep‐learning analysis to construct 3D phase diagrams across composition–temperature space within 30 min. We demonstrate a dual‐scale strategy that enables broad‐range exploration of phase space and localized refinement of critical transition boundaries. Meanwhile, tunable tri‐nodal temperature control at 25°C, 37°C, and 45°C provides a structured thermal dimension for systematically evaluating temperature‐dependent phase behavior. A VGG‐16–based four‐class classifier further distinguishes two‐phase condensates, single‐phase states, boundary states, and aggregate‐like phenotypes, achieving approximately 99.3% accuracy on an independent test set of 720 images. Applied to heparin sodium–mediated condensate modulation, T‐PhaseMap revealed a 17.6% temperature‐dependent variation in apparent suppression potency across 25°C–45°C. This platform provides a rapid and objective tool for physiologically relevant phase‐diagram generation and high‐throughput screening of condensate‐targeting therapeutics.

## Graphical abstract

By merging centrifugal microfluidics, precise temperature control, and AI image recognition, T‐PhaseMap transforms biomolecular condensate phase mapping into a rapid, temperature‐resolved workflow. It generates 3D composition–temperature phase diagrams within 30 min, resolves four phase states, and uncovers temperature‐dependent heparin sodium suppression, offering a practical route toward physiologically relevant condensate analysis and therapeutic screening.

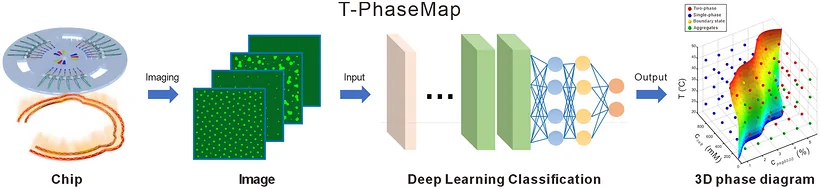

## 1 Introduction

Liquid–liquid phase separation (LLPS) of proteins and nucleic acids has emerged as a fundamental organizing principle in cell biology [1, 2, 3, 4], driving the formation of membraneless organelles including stress granules, P‐bodies, and nucleoli [5, 6, 7]. These biomolecular condensates concentrate specific components to create biochemical reaction centers and regulate diverse cellular processes from gene expression to stress responses [8, 9, 10, 11, 12]. Aberrant phase separation is increasingly recognized in disease pathology, with dysregulated condensate formation implicated in neurodegenerative diseases such as amyotrophic lateral sclerosis (ALS) and Alzheimer's disease, as well as cancer progression [13, 14, 15, 16, 17].

Central to decoding these complex biological processes is the ability to predict when and how phase separation emerges under defined physicochemical conditions [18]. Phase diagrams address this need by providing a quantitative map of phase behavior, mapping phase boundaries across composition space, and enabling systematic comparisons among conditions. Yet temperature is rarely treated as a primary experimental axis: many studies are conducted at room temperature (25°C), even though physiological functions occur at 37°C, and many disease contexts involve elevated temperatures (e.g., fever and inflammation). Temperature can shift phase boundaries and even induce reversible, stress‐responsive condensate formation. Therefore, neglecting the impact of temperature may obscure key biology [19, 20, 21, 22, 23, 24]. Consequently, the inability to efficiently interrogate temperature as an experimental dimension limits physiologically relevant insight and may yield misleading assessments of therapeutic efficacy when translating in vitro observations to in vivo settings [25, 26].

Recent microfluidic innovations are overcoming bottlenecks in throughput during phase‐diagram mapping. Li et al. developed a microfluidic platform for rapid determination of biomolecular liquid–liquid phase separation (LLPS) phase diagrams, which can generate up to 1750 concentration conditions within 140 min [27]. Villois et al. developed a label‐free droplet microfluidic framework, which measures dense‐phase volume fractions in finite volumes, enabling extraction of complete LLPS phase diagrams and kinetics [28]. Subsequently, Arter et al. developed PhaseScan, a droplet‐based microfluidic platform that generates picoliter‐scale compartments at high throughput, enabling substantial reductions in sample consumption [29]. Moving further toward data‐efficient discovery, Jansen et al. developed PhaseXplorer, an active‐learning framework that leverages Gaussian process regression to guide experiments, enabling four‐dimensional phase‐diagram reconstruction [30]. Despite multiple advances, no existing platform integrates temperature control as a true experimental variable while maintaining the throughput advantages of microfluidic automation and the objectivity of artificial intelligence‐based analysis.

Here, we report the temperature‐resolved phase‐mapping platform (T‐PhaseMap), an AI‐powered platform that charts phase behavior across composition–temperature space. T‐PhaseMap integrates (i) centrifugal concentration‐gradient generation, (ii) a tri‐nodal temperature‐control architecture, and (iii) AI‐enabled image‐based phase classification, enabling rapid, high‐fidelity monitoring of phase‐separation dynamics. This integration facilitates the autonomous construction of high‐density 3D phase matrices, offering a dual‐scale capability, which includes coarse‐grained screening across expansive parameter spaces and fine‐grained interrogation of UCST/LCST‐like transition boundaries (e.g., upper/lower critical solution temperature (UCST/LCST)‐like behavior). The platform compresses the complete workflow for generating temperature‐resolved 3D phase diagrams within 30 min and reveals significant temperature‐dependent drug effects on phase separation; this capability is experimentally validated using the clinical anticoagulant heparin sodium. Building on this platform, we further demonstrate temperature‐dependent modulation of biomolecular phase separation by heparin sodium, highlighting the potential of T‐PhaseMap for physiologically relevant drug screening.

## 2 Results

### 2.1 Platform Design and Operation

**FIGURE 1:**
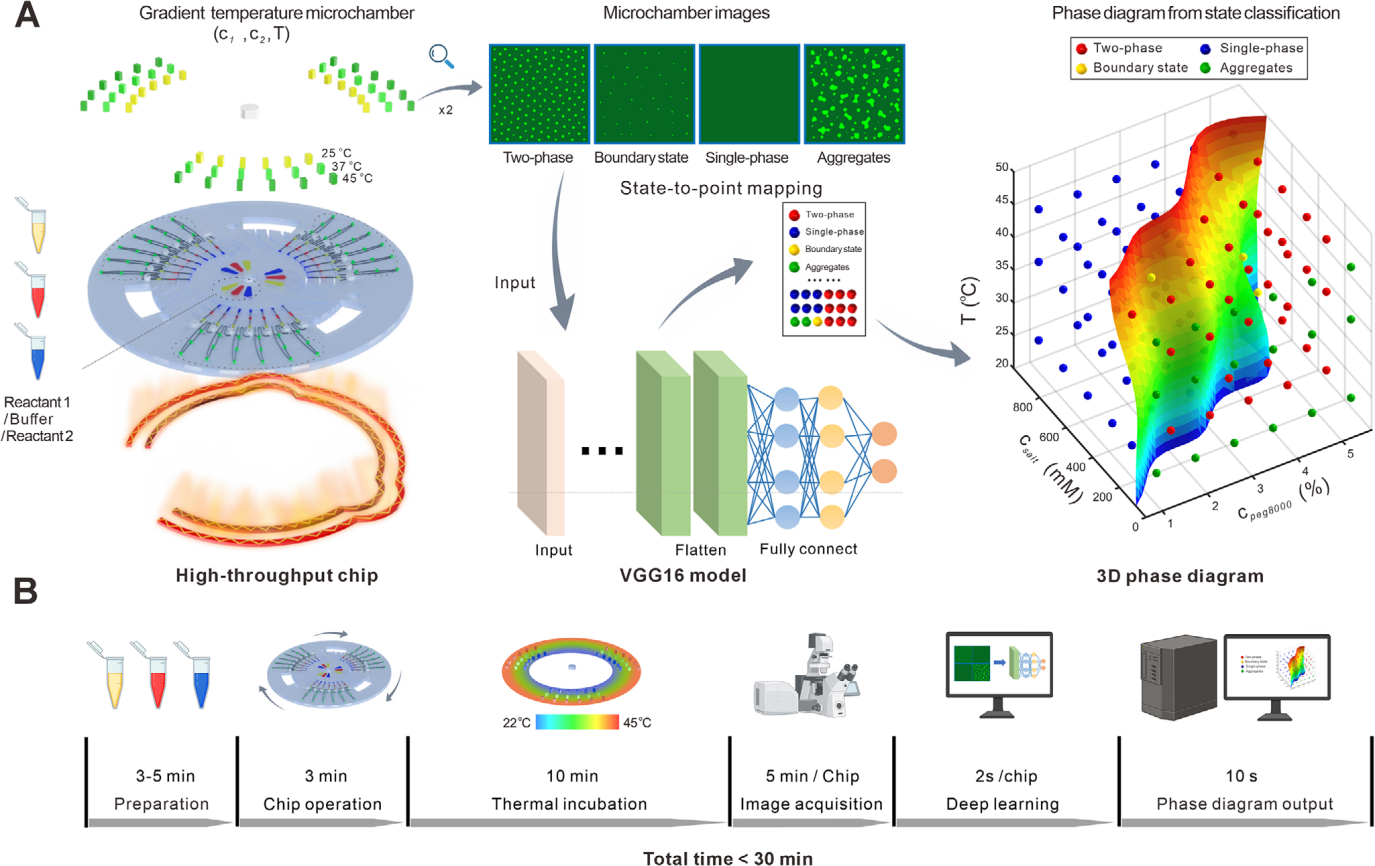
Overview of the T‐PhaseMap for AI‐powered, high‐throughput 3D phase‐diagram generation. (A) The T‐PhaseMap integrates programmed centrifugal actuation, performed using an automated centrifugal microfluidic device, with tri‐nodal temperature control provided by a standalone external temperature‐control module. Microchamber images exhibiting two‐phase, single‐phase, boundary state, or aggregate‐like phenotype are input to a VGG‐16 model for four‐class phase‐state classification. The classified data points are then assembled into 3D phase diagrams. (B) Six‐step workflow completed in <30 min.

The T‐PhaseMap architecture combines an automated centrifugal microfluidic device, a standalone external temperature‐control module, and a deep‐learning‐assisted analysis pipeline, enabling autonomous construction of multidimensional phase diagrams (Figure 1A). Centrifugal actuation provided by the automated device drives the formation of well‐defined concentration gradients within the chip, while the standalone external temperature‐control module establishes three adjustable temperature setpoints selected from a range of temperatures (e.g., 25°C, 31°C, 37°C, 41°C, and 45°C), enabling highly parallel evaluation of phase behavior across temperatures for each composition. Together, these controls enable high‐fidelity capture of temperature‐responsive features and rapid localization of thermal transition regions (e.g., UCST/LCST‐like boundaries) within the explored parameter space. As detailed in Figures S1–S3, the programmable centrifugal actuation, closed‐loop temperature‐control module, and inverted chip–heater fluorescence‐imaging configuration were systematically validated. The centrifugal protocol enabled sequential sample loading, primary distribution, vibration‐assisted mixing, and final dispensing into the gradient‐temperature microchambers (Figure S1). The annular resistive‐heater module provided stable closed‐loop thermal regulation at the primary experimental temperature nodes of 25°C, 37°C, and 45°C, and was additionally calibrated at 31°C and 41°C for intermediate‐temperature validation experiments (Figure S2). During fluorescence imaging, the chip was mounted in an inverted configuration on the external temperature‐control module, with the chip positioned at the bottom for in situ microscopy under thermal regulation (Figure S3).

To improve throughput and reduce the time cost of manual inspection, raw fluorescence images acquired from individual microchambers were fed into a VGG‐16 engine for four‐class phase‐state classification. The model distinguished two‐phase condensates, homogeneous single‐phase states, boundary states, and aggregate‐like phenotypes, thereby enabling more refined and objective annotation of condensate phase behavior. The resulting digitized labels are projected back into the concentration–temperature coordinates via a state‐to‐point mapping procedure to form dense, matrix‐style phase maps. Continuous 3D phase‐diagram surfaces are then reconstructed using a support vector machine (SVM) with a radial basis function (RBF) kernel. Together, this synergy enables multi‐resolution phase‐diagram construction, supporting both wide‐range boundary screening and fine interrogation of selected concentrations. This technology also compresses the end‐to‐end workflow to < 30 min (Figure 1B), including sample preparation, gradient generation, thermal incubation, image acquisition, and computational rendering.

### 2.2 Dual‐Scale Strategy and Scalable 3D Phase‐Diagram Construction

**FIGURE 2:**
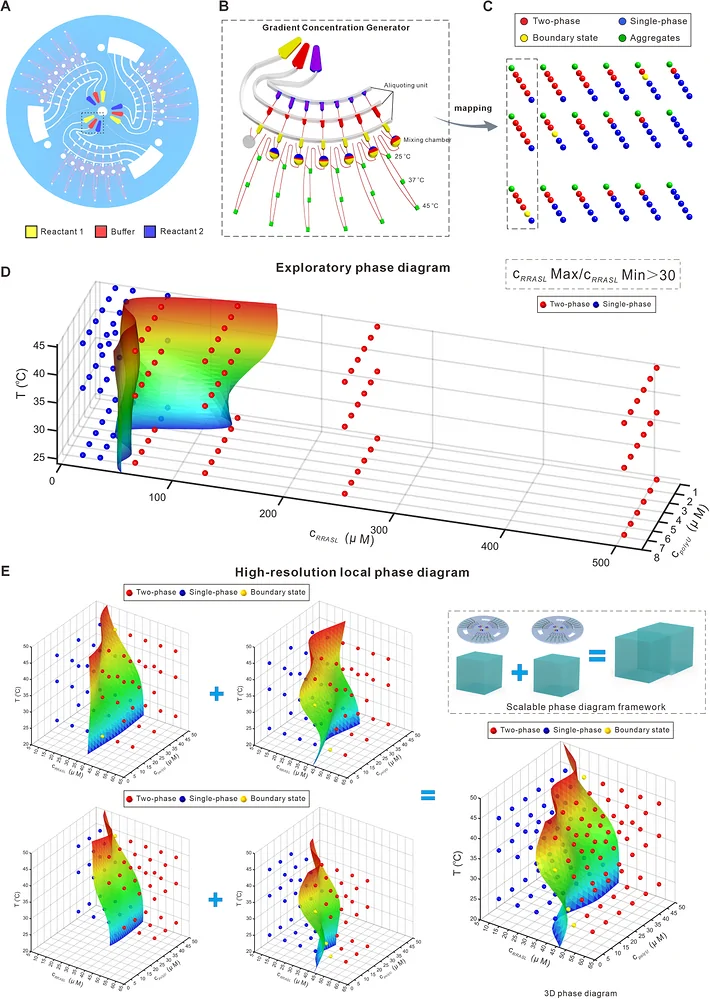
High‐throughput microfluidic platform and the scalable construction of 3D phase diagrams. (A) Overview of the centrifugal microfluidic chip. (B) Illustration of the Gradient Concentration Generator. The unit integrates aliquoting and mixing chambers to produce precise concentration gradients. (C) The four‐class phase‐state labels output by the VGG‐16 classifier are plotted as discrete points in the 3D phase diagram within the (*c_1_*, *c_2_*, T) parameter space, enabling visualization of two‐phase condensates, single‐phase states, boundary states, and aggregate‐like phenotypes. (D) Exploratory phase diagram. Broad‐range sparse sampling (*c_RRASL_* up to 503 µm, dynamic range > 30) is used to rapidly localize global phase‐transition boundaries. (E) Scalable 3D phase‐diagram framework. High‐resolution local sampling is performed near critical boundaries to generate detailed 3D sub‐diagrams. These localized modules can be superimposed and integrated (indicated by “+” and “ = ” signs) to construct a comprehensive and high‐fidelity representation of the phase landscape.

As a well‐established model of electrostatically driven complex condensates (charge‐mediated LLPS), the polyU/RRASLRRASLRRASL repeat peptide system readily forms liquid‐like condensates. To quantitatively chart its phase behavior as a function of compositions (*c_polyU_* and *c_RRASL_*) and temperature, we built a high‐throughput microfluidic chip (Figure 2A). Fluorescence recovery after photobleaching (FRAP) measurements and droplet fusion assays consistently supported the liquid‐like properties of the polyU/RRASLRRASLRRASL condensates (Figure S4).

The platform enables controlled loading of three primary inputs (Reactant 1, Buffer, and Reactant 2) into an integrated Gradient Concentration Generator (GCG) (Figure 2B); the chip comprises three such GCG units. Within each unit, automated aliquoting and mixing generate prescribed concentration gradients, which are then centrifugally dispensed into the gradient‐temperature microchamber array. Notably, each on‐chip GCG unit generates a continuous concentration gradient, which is sampled at six concentration levels and evaluated at three temperature setpoints, yielding 18 concentration–temperature conditions for constructing the 3D phase diagram (Figure 2C). Importantly, the dynamic range and resolution of the concentration space can be flexibly tuned at the sample‐preparation stage: by preparing Reactant 1 (concentration *c_1_*) at either a highly separated or a closely spaced set of initial concentrations, the platform can switch between expansive screening and fine local interrogation without altering the chip layout. In contrast, Reactant 2 (concentration *c_2_*) requires only a single stock concentration, as its effective gradient magnitude is intrinsically determined by the microfluidic architecture. This synergy allows the platform to bridge the gap between broad screening and high‐resolution characterization, ensuring precise identification of phase boundaries during automated profiling. Combined with an external temperature‐control module, this design enables concurrent exploration of biomolecular phase space and temperature. Detailed structural and dimensional drawings of the centrifugal microfluidic chip, including the GCG geometry and the relative volume ratio of the six gradient microchambers in each GCG unit, are provided in Figure S5. The multilayer chip assembly, including the top cover, chamber layer, fluidic channel layer, and bottom cover, is shown in Figure S6.

The 3D phase diagram is constructed using a scalable, two‐stage strategy that balances breadth and precision. First, an exploratory phase diagram (Figure 2D) is generated over a broad concentration range (e.g., *c_RRASL_* up to 503 µm with a dynamic range > 30) to rapidly localize the global phase‐transition boundary. Guided by these coarse coordinates, high‐resolution local phase diagrams (Figure 2E) are then produced by densifying sampling around boundary regions to sharpen the transition contour. Multiple localized 3D data blocks can be seamlessly integrated into a unified phase surface, yielding a comprehensive yet high‐fidelity representation of the phase landscape. This modular workflow not only improves boundary localization efficiency but also provides an extensible route for systematically interrogating complex biomolecular condensate assembly under coupled composition–temperature perturbations.

### 2.3 Deep Learning Enables Rapid and Automatic Phase State Classification

T‐PhaseMap integrates a VGG‐16–based image‐classification engine that recognizes fluorescence microchamber images within a four‐class phase‐state framework, classifying them into two‐phase condensates, homogeneous single‐phase states, boundary states, and aggregate‐like phenotypes. Representative images from the polyU/RRASLRRASLRRASL and Krt1 systems illustrate these phenotypes, including boundary states and irregular aggregate‐like structures (Figure S7). FRAP analysis of GFP‐Krt1 condensates and aggregate‐like phenotypes further supported their distinction, with droplet‐like condensates showing clear fluorescence recovery after photobleaching, whereas aggregate‐like structures displayed substantially weaker recovery, indicating reduced molecular mobility (Figure S8).

To support the phenotypic definitions quantitatively, we extracted morphology‐ and intensity‐related descriptors from all classified images, including condensate count, condensate area, equivalent diameter, coefficient of variation, and partition coefficient K (Figure S9). Pairwise comparisons among the four phase‐state groups were performed using two‐sided Mann–Whitney U tests with Holm correction for multiple comparisons, and all adjusted *p* values were < 0.0001 (Table S1). These descriptors showed clear differences among the four condensate states, supporting the use of a refined four‐class classification framework rather than subjective visual assignment alone.

**FIGURE 3:**
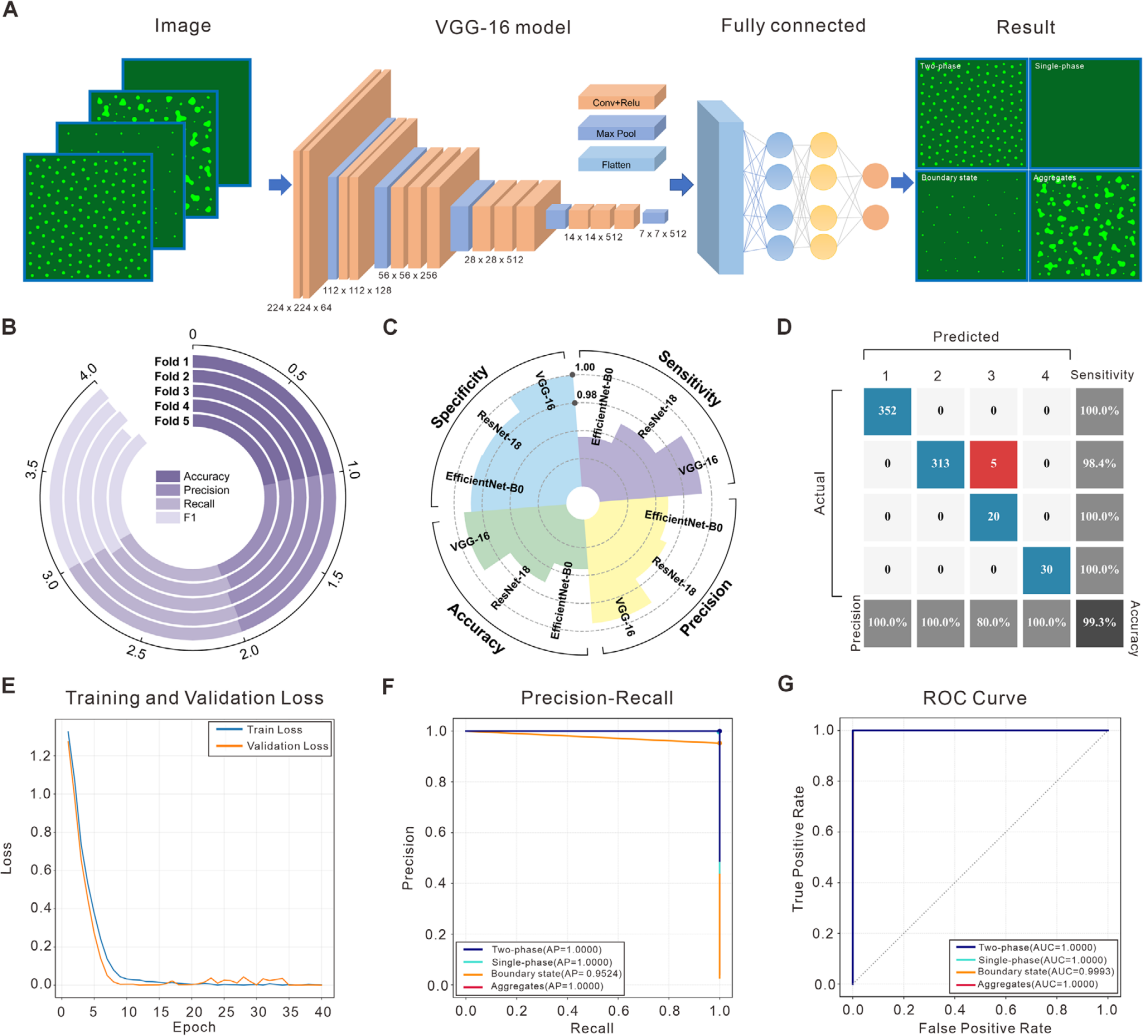
Performance evaluation of the VGG‐16 model for four‐class phase‐state recognition. (A) CNN architecture for classifying fluorescence images into four phenotypic states: two‐phase, single‐phase, boundary state, and aggregate‐like phenotype. (B) Fivefold cross‐validation performance of the VGG‐16 model, showing accuracy, precision, recall, and F1‐score for each fold. (C) Comparative performance of VGG‐16, ResNet‐18, and EfficientNet‐B0 based on accuracy, support‐weighted precision, support‐weighted sensitivity, and support‐weighted specificity. (D) Test‐set confusion matrix of the VGG‐16 model for four‐class classification. Labels 1–4 denote two‐phase images (*n* = 352), single‐phase images (n = 318), boundary‐state images (n = 20), and aggregate‐like phenotype images (n = 30), respectively. (E) Training and validation loss curves of the VGG‐16 model over 40 epochs (F) Precision–recall curves for the four classes. (G) Multi‐class ROC curves for the four classes.

The VGG‐16 model was then trained and evaluated under this four‐class phase‐state framework (Figure 3A). Fivefold cross‐validation demonstrated consistently high accuracy, precision, recall, and F1‐score across data partitions, indicating stable model performance (Figure 3B). The corresponding training and validation loss curves further showed stable convergence across folds (Figure S10). To evaluate whether VGG‐16 was appropriate for this task, we compared its performance with two alternative backbones, ResNet‐18 and EfficientNet‐B0. Under the present dataset, training strategy, and evaluation procedure, VGG‐16 exhibited earlier stabilization of the training and validation losses and achieved higher test‐set classification metrics than ResNet‐18 and EfficientNet‐B0 (Figure 3C and Figure S11). On an independent test set containing 720 fluorescence images, including two‐phase condensates (n = 352), single‐phase states (n = 318), boundary states (n = 20), and aggregate‐like phenotypes (n = 30), the VGG‐16 model achieved an overall accuracy of approximately 99.3%. The confusion matrix showed that only five single‐phase images were misclassified as boundary states, whereas the other three classes were correctly classified (Figure 3D). Training and validation loss curves of the selected VGG‐16 model also converged rapidly within 40 epochs, indicating stable optimization without evident overfitting (Figure 3E). The model further achieved high precision–recall and ROC performance (Figure 3F,G).

### 2.4 Temperature‐ and PEG8000‐Dependent Phase Behavior of Krt1 Condensates

To further evaluate the generalizability of T‐PhaseMap beyond the polyU/RRASLRRASLRRASL model system, we next applied the platform to a biologically relevant protein condensate system based on full‐length mouse Krt1. Krt1 was selected from our phase‐separation protein database, which was constructed based on our previously developed method [12]. Krt1 is closely associated with epidermal structure, skin barrier formation, and regulation of inflammatory responses. In addition, previous studies have shown that Krt1/K1 is upregulated in triple‐negative breast cancer tumors and can serve as a cell‐surface receptor for tumor‐targeted peptide–drug delivery [31]. Therefore, compared with the relatively simple peptide–RNA condensate model, Krt1 more closely represents a complex endogenous protein assembly system and is better suited for evaluating the applicability of T‐PhaseMap to protein phase‐behavior analysis. The Krt1 system exhibited more diverse image‐based phenotypes, including droplet‐like two‐phase condensates, near‐boundary states, homogeneous single‐phase states, and irregular aggregate‐like phenotypes (Figure S7).

**FIGURE 4:**
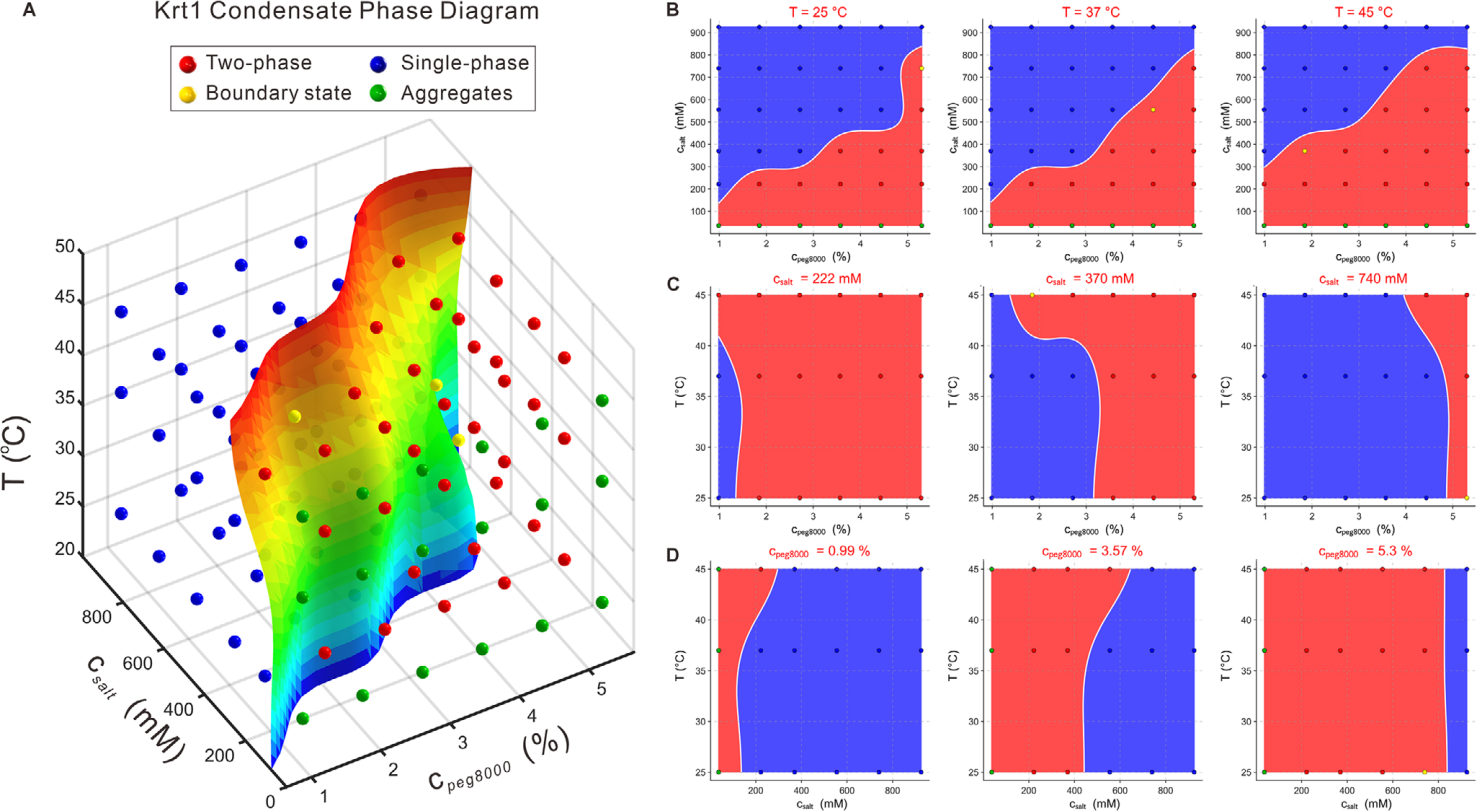
3D phase diagram of the Krt1 system across salt concentration, PEG8000 (% w/v) concentration, and temperature. The salt concentration was defined as 50 mm Tris‐HCl plus NaCl; therefore, NaCl concentration was adjusted to achieve the indicated total salt concentration. (A) 3D phase diagram of the Krt1 system across salt concentration, PEG8000 concentration, and temperature. (B) 2D phase maps at fixed temperatures of 25°C, 37°C, and 45°C. (C) T–PEG8000 cross‐sections at fixed salt concentrations. (D) T–salt cross‐sections at fixed PEG8000 concentrations.

We then constructed a temperature‐ and PEG8000‐dependent phase diagram of Krt1 condensates at a fixed Krt1 concentration of approximately 2.43 µm, with phase states mapped as a function of salt concentration, PEG8000 concentration (% w/v), and temperature (Figure 4A). The extracted 2D phase maps at fixed temperatures revealed that Krt1 phase behavior was jointly regulated by molecular crowding, ionic strength, and temperature (Figure 4B). Temperature‐resolved cross‐sections further showed that Krt1 condensation became more favorable at elevated temperatures within the examined composition window, displaying an LCST‐like tendency. In contrast, increasing salt concentration tended to suppress droplet formation and shifted the system toward the single‐phase state, suggesting that the ionic environment modulates Krt1 condensate formation (Figure 4C). Notably, under excessively low‐salt conditions, Krt1 preferentially formed an aggregate‐like phenotype rather than liquid‐like condensates, highlighting the need to distinguish aberrant aggregation from canonical liquid–liquid phase separation. At a fixed temperature, increasing PEG8000 concentration generally promoted condensate formation and expanded the two‐phase region, consistent with crowding‐enhanced assembly of protein condensates (Figure 4D).

These results indicate that Krt1 phase behavior cannot be described by a single compositional variable, but instead emerges from the coupled effects of temperature, salt concentration, and molecular crowding. Collectively, the Krt1 validation demonstrates that T‐PhaseMap is applicable not only to simplified polymer‐ or peptide‐based condensate models, but also to more biologically relevant protein systems with complex and heterogeneous phase‐state phenotypes.

### 2.5 Temperature‐Dependent Drug Effects on Phase Separation

Heparin sodium is a clinically used, highly sulfated glycosaminoglycan and a strong polyanion, making it a convenient model perturbant for electrostatically driven peptide–RNA condensates.

To demonstrate our platform's ability to quantify drug‐induced remodeling of condensate phase landscapes, we chose heparin sodium as a model perturbant (concentrations denoted as *c_hep_*) and added it to pre‐formed polyU/RRASLRRASLRRASL condensates, mapping phase outcomes across temperature and composition space. Condensates were prepared at a fixed stoichiometric ratio (*c_RRASL_*/*c*_polyU_ ∼9.2); therefore, *c_tot_* is reported using the nominal *c_RRASL_* value (i.e., *c_tot_* is approximated as *c_RRASL_*) for simplicity.

**FIGURE 5:**
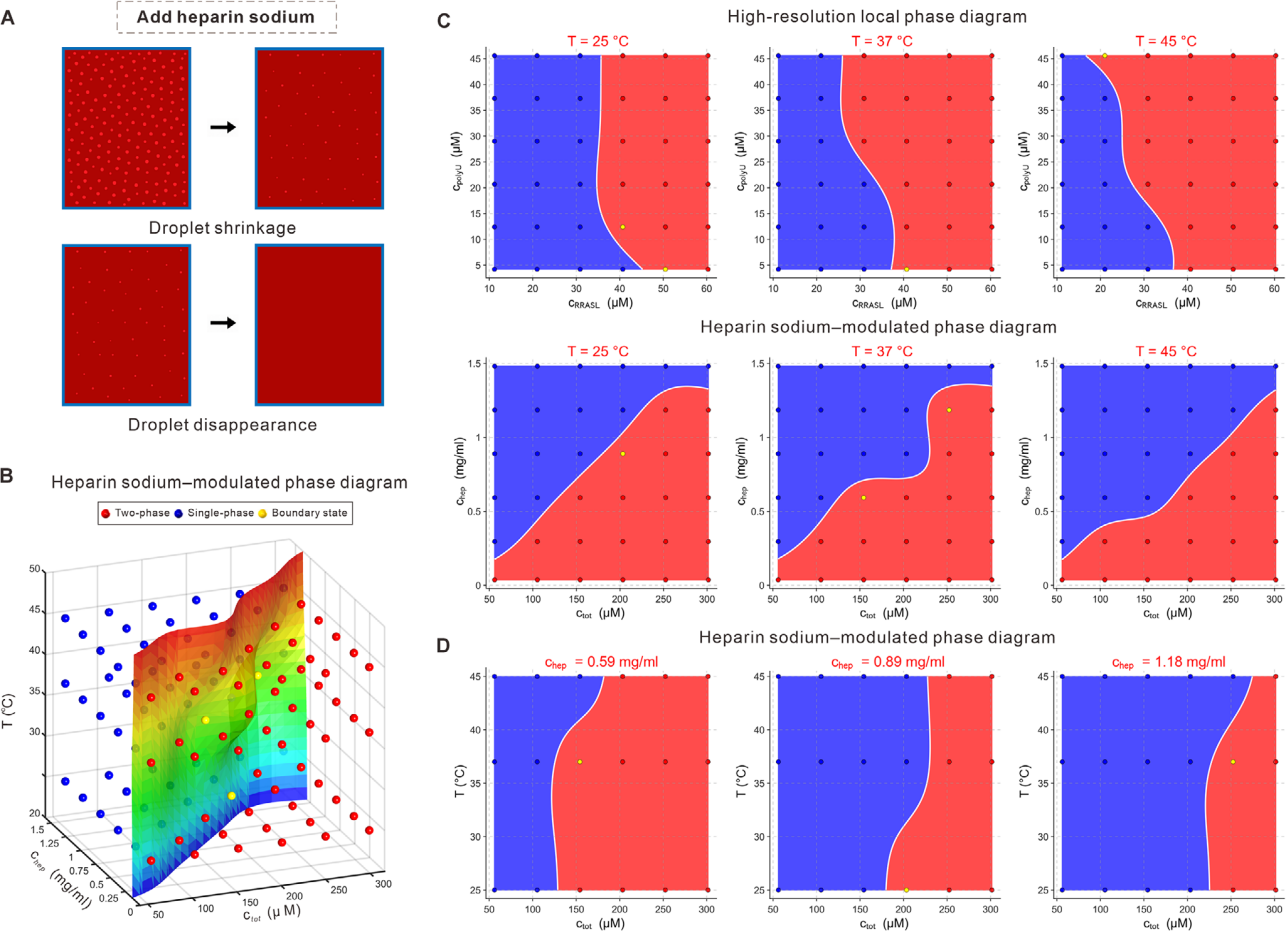
Heparin sodium suppresses polyU/RRASLRRASLRRASL phase separation with a pronounced temperature dependence. (A) Schematic illustration showing droplet shrinkage and eventual disappearance after adding heparin sodium. (B) Heparin sodium–modulated 3D phase diagram illustrating temperature‐dependent changes in phase separation. (C) 2D phase maps at 25°C, 37°C, and 45°C, including the high‐resolution local *c_polyU_*–*c_RRASL_* binary interaction map and heparin sodium‐involved ctot–chep maps. (D) T–*c_tot_* phase‐map slices at fixed *c_hep_* for the heparin sodium‐regulated polyU/RRASLRRASLRRASL system.

As illustrated schematically in Figure 5A, the addition of heparin sodium caused a pronounced decrease in droplet size and, at sufficiently high heparin sodium levels, complete droplet dissolution. Representative fluorescence micrographs confirmed this heparin sodium–induced droplet dissolution behavior (Figure S12). This macroscopic “droplet disappearance” suggests that heparin sodium weakens the effective interactions that stabilize the dense phase, thereby contracting the two‐phase region and shifting the phase boundary.

We next summarized the multidimensional phase outcomes in a heparin sodium–modulated 3D phase diagram spanning temperature (T), total solute concentration *c_tot_*, and heparin sodium concentration *c_hep_* (Figure 5B). Experimentally classified single‐phase and two‐phase states form a well‐defined boundary surface, providing an intuitive map of the LLPS stability window under heparin sodium perturbation.

To quantitatively compare heparin sodium efficacy across temperatures, we further operationally defined a critical suppression concentration at each (*c_tot_*,*T*). Specifically, for a given (*c_tot_*,*T*), we ordered measurements by increasing *c_hep_* and identified the first condition at which the system became single‐phase (blue label); the corresponding concentration was defined as:

(1) $$c_{\mathrm{hep},\mathrm{crit}}\left(c_{\mathrm{tot}},T\right)=\min\left\{c_{hep}\left|\mathrm{single}\;\mathrm{phase}\right.\right\}$$

This analysis yields a discrete critical boundary, *c*_hep, crit_(*c*_tot_, *T*), for each temperature slice. To obtain a single temperature‐specific index, we computed the *c_tot_*‐weighted mean critical concentration over the sampled *c_tot_* range,

(2) $${\bar{c}}_{\mathrm{hep},\mathrm{crit}}\left(T\right)=\frac{\int c_{\mathrm{hep},\mathrm{crit}}\left(c_{\mathrm{tot}},T\right)\;dc_{\mathrm{tot}}}{c_{\mathrm{tot},\max}-c_{\mathrm{tot},\min}}$$

Under this definition, a smaller ${\bar{c}}_{\mathrm{hep},\mathrm{crit}}\left(T\right)$ indicates that less heparin sodium is required to suppress phase separation, corresponding to stronger overall efficacy. Based on our calculated ${\bar{c}}_{\mathrm{hep},\mathrm{crit}}\left(T\right)$‐derived potency metric, we observe pronounced temperature‐dependent drug effects: heparin sodium exhibits a 17.6% variation in condensate‐modulating potency across the 25–45°C thermal window, highlighting the necessity of temperature‐resolved pharmacological assays.

Importantly, 2D slices extracted from this 3D landscape reveal distinct temperature responses depending on whether heparin sodium is present. In the high‐resolution local binary interaction map (*c_polyU_*–*c_RRASL_*, Figure 5C, top), elevating temperature expands the two‐phase region and shifts the boundary toward lower *c_RRASL_*, consistent with an LCST‐like tendency in the native polyU/RRASLRRASLRRASL interaction network (i.e., phase separation becomes easier upon heating within the probed composition window).

By contrast, in the heparin sodium‐containing *c_tot_*–*c_hep_* maps (Figure 5C, bottom), the phase boundary is markedly reshaped as temperature increases, with the single‐phase region becoming progressively favored; this implies a transition toward a UCST‐like tendency. This indicates that the heparin sodium‐induced suppression effect exhibits a pronounced temperature dependence and becomes more prominent at elevated temperatures.

Finally, T–*c_tot_* cross‐sections at fixed *c_hep_* (Figure 5D) further support the inhibitory role of heparin sodium: increasing *c_hep_* systematically expands the single‐phase region across a broad concentration range. Collectively, these results identify heparin sodium as an effective suppressor of polyU/RRASLRRASLRRASL LLPS and also suggest that its inhibitory effect is temperature dependent.

## 3 Discussion

Here, we report T‐PhaseMap, an AI‐powered, temperature‐resolved phase‐mapping platform that characterizes phase behavior across composition–temperature space and addresses a key gap in biomolecular phase‐separation research: the lack of physiologically relevant phase‐diagram generation beyond conventional room‐temperature workflows. The platform addresses key bottlenecks in conventional phase separation characterization, including labor‐intensive preparation, limited ability to treat temperature as an experimental axis, subjective visual scoring, and long turnaround times for multidimensional mapping.

This platform demonstrates unique advantages. First, T‐PhaseMap explicitly incorporates temperature as an experimental variable through a tri‐nodal temperature‐control architecture (25°C, 37°C, and 45°C) integrated with centrifugal gradient, enabling parallel assessment of phase behavior across multiple temperatures within a single run. Second, on an independent test set containing 720 images, the VGG‐16 model achieved an overall accuracy of approximately 99.3%. Precision–recall and ROC analyses further indicated high classification performance. Third, the end‐to‐end workflow, from gradient generation and thermal incubation to imaging and computational rendering, can be completed in <30 min, substantially compressing the phase‐diagram generation cycle. To further contextualize these advantages, we added Table S2, which quantitatively compares T‐PhaseMap with existing platforms in terms of throughput, temperature control, sample consumption, end‐to‐end time, and phase‐state classification strategy.

The platform enables thermally resolved insights that are difficult to obtain using room‐temperature‐only assays. Many existing phase diagrams are generated at a single, often non‐physiological temperature (typically room temperature). Instead, T‐PhaseMap mitigates this by mapping phase behavior across discrete temperature nodes in the same experiment. In a model polyU/RRASLRRASLRRASL system, we show that heparin sodium is a potent suppressor of LLPS and that its effect is temperature dependent: the phase boundary is markedly reshaped with temperature, and the impact of heparin sodium shows ∼17.6% variation in its condensate‐modulating potency, as reflected by the boundary concentration between 25°C and 45°C, highlighting the need for thermally resolved pharmacological assays.

Overall, T‐PhaseMap provides a fast, efficient, and intuitive approach to mapping condensate phase behavior across coupled composition–temperature space. One current limitation is that the 3D phase diagrams are reconstructed from experimentally classified phase states at discrete temperature nodes. Therefore, the RBF‐SVM–reconstructed surfaces should be interpreted as computational visualizations of interpolated decision boundaries within the sampled composition–temperature space, rather than as direct evidence of intrinsic thermodynamic continuity. To assess the reliability of this interpolation, we performed additional validation experiments at two intermediate temperatures, 31°C and 41°C, for the Krt1 condensate phase diagram, the high‐resolution local phase diagram, the heparin sodium–modulated phase diagram, and the exploratory phase diagram. We first compared the RBF‐SVM–reconstructed 3D phase diagrams generated with and without incorporating the experimentally measured phase‐state data at 31°C and 41°C into the RBF‐SVM fitting (Figure S13). We then directly compared the experiment‐informed and RBF‐SVM–interpolated 2D phase maps at 31°C and 41°C (Figure S14). Quantitative comparison between the phase‐state classifications generated by the original RBF‐SVM model and the corresponding experimentally measured LLPS/non‐LLPS labels showed classification accuracies ranging from 94.44% to 98.61% across the four phase‐diagram datasets (Table S3). The mismatches observed in Figure S14 were concentrated near the phase boundaries, indicating local discrepancies in the interpolated boundary positions. These deviations may arise from the limited temperature sampling used for the original RBF‐SVM fitting, because smooth decision surfaces reconstructed from discrete experimental phase‐state labels may not fully capture local variations of the phase boundaries with temperature and composition. Accordingly, the reconstructed surfaces should be regarded as approximate computational representations of the overall phase landscape rather than substitutes for direct experimental measurements. Specific phase states and precise boundary locations under conditions of interest require experimental verification. Extrapolation beyond the sampled parameter range should therefore be avoided, and denser temperature sampling will be required when sharp, non‐monotonic, or near‐boundary thermal transitions are expected.

Future developments will focus on increasing temperature sampling density through higher‐throughput chip architectures and spatially programmable thermal‐control systems, enabling continuously temperature‐resolved phase‐diagram generation with minimized thermal cross‐talk. In addition, although image‐based multi‐class classification can recognize aggregate‐like phenotypes, static fluorescence images alone cannot fully resolve the intrinsic material states of condensates. We therefore anticipate that integrating multi‐class phenotype readouts with dynamic measurements, such as fluorescence recovery after photobleaching (FRAP) and time‐series imaging, will enable quantitative assessment of condensate fluidity and rigidity, thereby improving discrimination among liquid‐like phase‐separated droplets, gel‐like condensates, and pathological aggregates.

## 4 Experimental Section

### 4.1 Fabrication of the Microfluidic Chip

Microchannel and chamber geometries of the T‐PhaseMap were drafted in AutoCAD (Autodesk, Inc., Mill Valley, CA) and produced on polymethyl methacrylate (PMMA) sheets (YongChip, China) using CO_2_ laser cutting (Laser Technology, China) through a rapid printing–cutting–lamination (PCL) workflow. Guided by alignment posts, the patterned layers were stacked sequentially from bottom to top and then thermally bonded at 50°C for 10 min (Hengwei Precision Technology, China) to yield the fully integrated chip.

### 4.2 Reagents and Materials

Polyuridylic acid potassium salt was purchased from Aladdin (P350073), and phosphate‐buffered saline (PBS) from Thermo Fisher Scientific (10010023). Heparin sodium was purchased from MedChemExpress (HY‐17567A). The peptide RRASLRRASLRRASL and its *N*‐terminally 5‐carboxytetramethylrhodamine (TAMRA)–labeled analogue were custom synthesized by Sangon Biotech and supplied as trifluoroacetate salts. All reagents were dissolved in ultrapure water (resistivity ≥ 18.2 MΩ cm) obtained from a Genie PURIST purification system.

### 4.3 Confocal Fluorescence Imaging

Fluorescence images were acquired on an Olympus Fluoview FV3000 confocal laser scanning microscope equipped with an IX83 fully motorized inverted microscope (Olympus). All images were collected using a 10× objective lens. Acquisition settings were kept consistent across samples within each experiment.

### 4.4 Temperature‐Control Platform and Closed‐Loop Regulation

Temperature control of the centrifugal microfluidic chip was achieved using an external heating and control module. An annular resistive heater was integrated beneath the chip and connected to an adjustable temperature controller and a DC power supply to form the heating circuit. The annular resistive heater and the temperature controller were purchased from Dongtai Kedeman Electric Heating Technology Factory, and the DC power supply (MN305C) was purchased from Huizhou Dayawan Aoteng Electronic Products Distributor. During operation, the controller adjusted the heater power according to real‐time feedback from the on‐chip temperature sensor(s), enabling closed‐loop temperature regulation and stable maintenance. All temperature tests were performed at an ambient temperature of ∼22°C.

### 4.5 Deep‐Learning Classification Models

We implemented an offline data augmentation strategy based on the largest inscribed square. Each original image was rotated from 0° to 355° in 5° increments. To prevent black padding introduced by rotation from interfering with feature learning, for each rotation angle we calculated the largest axis‐aligned square fully contained within the rotated image and then performed center cropping accordingly. To construct the development dataset for the four‐class model, 15 representative original fluorescence microscopy images were selected from each morphological category, including two‐phase, single‐phase, boundary states, and aggregate‐like phenotypes, yielding 60 original images in total. Each original image was processed using the largest‐inscribed‐square augmentation strategy, increasing the dataset size by a factor of 72 and generating 4320 augmented samples in total. This augmentation procedure was used to expand the effective training dataset and reduce overfitting under small‐sample conditions. To minimize the risk of data leakage and enable hyperparameter tuning, we performed five‐fold cross‐validation at the original‐image level, ensuring that all augmented samples derived from the same original image were strictly assigned to the same fold.

To accelerate convergence and leverage strong low‐level visual feature extraction, the VGG‐16 backbone network, as well as the ResNet‐18 and EfficientNet‐B0 comparator models, were initialized with weights pretrained on the ImageNet dataset. We then performed transfer learning by modifying the fully connected classification head to output four categories and training it from scratch, while fine‐tuning the pretrained convolutional layers on our fluorescence microscopy images. All input fluorescence microscopy images were first resized to 256 × 256 pixels and then randomly cropped to 224 × 224 pixels. Online data augmentation was further applied, including random horizontal flipping and color jittering within a ±20% range for brightness, contrast, and saturation. These augmentation operations were intended to reduce image‐view bias in the positions of visually observed condensates and to simulate illumination variations caused by excitation fluctuations and nonuniform fluorescent‐solution conditions during microscopy imaging.

The networks were optimized using the adaptive moment estimation (Adam) optimizer with a batch size of 16. A weight decay coefficient of 1 × 10^−4^ was used for L2 regularization, and Dropout was incorporated to further reduce overfitting. A cosine annealing learning‐rate schedule was applied during training, and the loss function was cross‐entropy loss with label smoothing, with the smoothing coefficient α set to 0.05. Because different CNN architectures exhibited distinct convergence characteristics, the training duration and learning‐rate settings were adjusted on an architecture‐specific basis. For VGG‐16, the model was trained for 40 epochs with the learning rate annealed from 1 × 10^−6^ to 1 × 10^−7^. For ResNet‐18, the maximum number of training epochs was extended to 80, with the learning rate annealed from 1 × 10^−6^ to 1 × 10^−7^. For EfficientNet‐B0, the maximum number of training epochs was also extended to 80, while the learning rate was annealed from 1 × 10^−5^ to 1 × 10^−6^. For each architecture, model performance was monitored on the validation set during training, and the checkpoint achieving the highest validation accuracy was selected as the final model for evaluation on the completely independent test set of 720 images, which were acquired from experiments, without any data augmentation.

### 4.6 RBF‐SVM–Based 3D Decision Surface Reconstruction and Visualization

A MATLAB workflow was used to reconstruct and visualize the 3D decision boundary based on the classified phase‐state labels. Coordinate data were imported from an Excel spreadsheet, and each data point was assigned a color according to its phase‐state phenotype: red for two‐phase condensates, yellow for boundary states, blue for single‐phase states, and green for aggregate‐like phenotypes. For binary decision‐boundary reconstruction, points classified as two‐phase, boundary‐state, or aggregate‐like phenotypes were assigned to the LLPS class, whereas points classified as single‐phase states were assigned to the non‐LLPS class.

The feature matrix was constructed from the spatial coordinates ([*x*, *y*, *z*]), and binary labels were assigned as 1 for phase‐separated samples and 0 for non‐phase‐separated samples. An RBF‐kernel SVM was trained using standardized predictors, automatic kernel‐scale estimation, a box constraint of 100, and sample weights, with yellow reference points assigned a threefold weight to strengthen their influence on the boundary.

The trained SVM was evaluated on a regular 3D grid covering the plotting domain. The positive‐class decision scores were reshaped into a scalar field, and the zero‐level isosurface ((f(x,y,z) = 0)) was extracted as the classification boundary. The resulting surface was rendered as a semi‐transparent Z‐dependent red–blue gradient patch, while sample points were displayed as shaded spheres. Interactive controls were included for sphere size, lighting, surface transparency, surface reflection, surface block size, and viewing angle adjustment.

### 4.7 Quantitative Image Analysis of Condensate Morphology

To quantitatively characterize condensate morphology and phase‐separation dynamics, an automated image‐analysis workflow was established based on fluorescence microscopy images. Raw microscopy images were first converted to grayscale images. Contrast‐limited adaptive histogram equalization (CLAHE) was then applied to enhance local contrast and improve the visibility of low‐contrast condensates, particularly under near‐boundary phase‐separation conditions. The images were subsequently denoised using a Gaussian smoothing filter with a 3 × 3 kernel. Condensates were segmented using local adaptive thresholding. This method calculates gray‐level thresholds within local image windows, thereby improving segmentation robustness under different condensate densities and spatially heterogeneous fluorescence backgrounds. Connected‐component analysis was then performed to identify individual condensates. Objects with an area smaller than 10 px^2^ were excluded as noise or segmentation artifacts. Based on the segmented condensate masks, multiple morphological and intensity‐related parameters were automatically extracted, including condensate number, area fraction, mean equivalent diameter, standard deviation of equivalent diameter, coefficient of variation (CV), area‐weighted roundness, and partition coefficient (K). The partition coefficient was calculated from the fluorescence‐intensity ratio between the condensate‐rich phase and the surrounding dilute phase. These parameters enabled high‐resolution quantitative characterization of condensate morphology across different phase states.

### 4.8 Prokaryotic Expression and Purification of Recombinant His‐GFP‐Krt1 Protein

For in vitro analysis of His‐GFP‐Krt1 protein expression, the coding sequence of His‐GFP‐Krt1 was cloned into the expression vector using the In‐Fusion cloning method. The resulting plasmid was transformed into chemically competent Escherichia coli BL21(DE3) cells. Transformants were cultured in Luria–Bertani (LB) medium supplemented with 0.05 mg/mL kanamycin and induced with 0.1 mm isopropyl β‐D‐1‐thiogalactopyranoside (IPTG) at 16°C overnight with shaking at 250 rpm.

The induced bacterial cells were harvested by centrifugation at 10 000 rpm for 15 min at 4°C. The cell pellet was resuspended in lysis buffer containing 50 mm Tris‐HCl, pH 8.0, 300 mm NaCl, and 30 mm imidazole, supplemented with an EDTA‐free protease inhibitor cocktail. After cell lysis, the lysate was clarified by centrifugation, and the supernatant was incubated with Ni‐NTA resin to capture the GFP‐Krt1‐His fusion protein. The resin was washed with lysis buffer, and the bound protein was eluted with elution buffer containing 50 mm Tris‐HCl, pH 8.0, 950 mm NaCl, 1000 mm imidazole, and 5% glycerol.

The eluted His‐GFP‐Krt1 protein was further purified by size‐exclusion chromatography using a Superdex 200 Increase column (Cytiva, Sweden). Fractions containing purified His‐GFP‐Krt1 were collected for subsequent analysis. All purification procedures were performed at 4°C.

## Author Contributions

**Yiwei Li**, **Peng Chen**, and **Bi‐Feng Liu** conceptualized the study, guided experimental design, analyzed data, revised the manuscript, and provided financial support. **Jiashuo Li** conceptualized the study, performed major experiments, and wrote the manuscript. **Pengjie Li** performed chip optimization and data analysis, participated in manuscript proofreading, and provided financial support. **Kaiting Zhang** conducted the deep‐learning analysis. **Yongqi Guo** built the external temperature‐control module.

## Conflicts of Interest

The authors declare no conflict of interest.

## Supporting information




**Supporting File**: advs77523‐sup‐0001‐SuppMat.docx.

## Data Availability

The AI model developed in this study, including the trained model weights and source code for model training, evaluation, prediction, and data augmentation, is freely available through our public GitHub repository: https://github.com/jiashuo‐li/T‐PhaseMap. Representative test images are also provided in the repository. Other data supporting the findings of this study are available from the corresponding author upon reasonable request.
