# Supplementary material for: AI‐Powered, Temperature‐Resolved Centrifugal Microfluidics for Rapid 3D Phase‐Diagram Generation of Biomolecular Condensates
Source: Adv Sci (Weinh). 2026 Aug 31:e77523. Online ahead of print. doi: 10.1002/advs.77523 (PMC13530018; doi:10.1002/advs.77523)
Supplement: Supplementary file 1 [file ADVS-9999-e77523-s001.docx]

# Supporting Information

# AI-powered, temperature-resolved centrifugal microfluidics for rapid 3D phase-diagram generation of biomolecular condensates

*Jiashuo Li§, Pengjie Li§, Kaiting Zhang§, Yongqi Guo, Peng Chen*, Bi-Feng Liu*, Yiwei Li**

This file includes

Figures S1–S14

Tables S1-S3

## Supporting Figures:

**Figure S1: Schematic and validation of the programmable, automated centrifugal microfluidic device.**

A. Portable centrifugal driving device and its program interface. The table on the right shows the automated centrifugation protocol.

B. Schematic illustration of on-chip fluid transport and aliquoting: (i) after sample loading, a pre-spin at 700 rpm for 30 s drives the sample into the central region; (ii) 2500 rpm for 20 s enables primary distribution of the sample along microchannels into individual branches; (iii) a vibration mode (200 rpm for 60 s) with reciprocating centrifugation within a 30° angular range promotes in-chamber mixing; (iv) a final spin at 1000 rpm for 20 s performs secondary distribution into the gradient temperature microchambers.

C. Representative photographs corresponding to steps (i–iv) in (B), demonstrating quantitative loading, barrier breakthrough, mixing, and multi-channel distribution of the liquid within the chip.

**Figure S2: External temperature-control module for the centrifugal microfluidic chip: setup and thermal performance.**

A. Photograph of the temperature-control setup integrated with the centrifugal microfluidic chip. An annular resistive heater is embedded beneath the chip and connected to an adjustable temperature controller and power supply.

B. Wiring diagram of the temperature-control circuit. The controller modulates heater power based on real-time feedback from on-chip temperature sensors, enabling closed-loop regulation.

C. Top view of the chip showing the annular heater layout and the temperature setpoints at different locations. In a 22°C ambient environment, the innermost chamber region is maintained at 25°C via heat transfer from the outer annular resistive heater.

D. Temperature–time responses at setpoints of 25°C, 31°C, 37°C, 41°C, and 45°C, demonstrating rapid heating and stable control (n = 3).

E. Infrared thermographs acquired at the corresponding setpoints, visualizing the surface temperature field across the chip; color scale: 20–50°C.

**Figure S3.** In situ fluorescence imaging with an inverted chip–heater assembly.

A. Photograph of the chip mounted on the external temperature-control module during microscopy imaging, with the assembly inverted (chip at the bottom).

B. Schematic of the imaging setup: a 561 nm laser is directed to the chip while the external temperature-control module (annular resistive heater) maintains the preset temperature.

**Figure S4:** **FRAP measurements and droplet fusion.**

A. FRAP of polyU/RRASLRRASLRRASL droplets.

B. Fusion of polyU/RRASLRRASLRRASL droplets.

**Figure S5**. Detailed structural and dimensional design of the centrifugal microfluidic chip.

A. Overall layout of the centrifugal microfluidic chip, showing the integrated arrangement of the Gradient Concentration Generator (GCG) units and the downstream gradient-temperature microchamber arrays. The outer diameter of the chip is 150 mm.

B. Enlarged view of a single GCG unit, illustrating the microchannels, aliquoting and mixing chambers, and downstream distribution channels used for concentration-gradient generation. Key structural dimensions are annotated in the drawing.

C. Schematic representation of the six gradient microchambers in each GCG unit, with their relative chamber-volume ratio specified as 4.10 : 3.43 : 2.77 : 2.10 : 1.43 : 0.76. All dimensions are given in millimeters, and Ø denotes diameter.

**Figure S6.** Exploded-view schematic of the multilayer chip. From top to bottom: top cover (0.4 mm), chamber layer (2.0 mm), fluidic channel layer (0.3 mm), and bottom cover (0.4 mm). These layers are aligned and assembled to form enclosed chambers and interconnected fluidic pathways.

**Figure S7.** Representative fluorescence microscopy images of the phase behaviors of two biomolecular systems. Scale bar, 200 μm.

A. Representative images of the polyU/RRASLRRASLRRASL system under different conditions, showing the two-phase state, boundary state, and single-phase state, respectively.

B. Representative images of the Krt1 system modulated by salt concentration and PEG8000 concentration, showing the two-phase state, boundary state, single-phase state, and aggregate-like phenotypes, respectively.

**Figure S8. FRAP analysis of GFP-Krt1 condensates and aggregate-like phenotypes.**
A. Representative FRAP images and recovery curve of liquid-like GFP-Krt1 condensates. Scale bar, 5 μm.

B. Representative FRAP images and recovery curve of aberrant GFP-Krt1 aggregate-like phenotype. Scale bar, 1 μm.

**Figure S9.** Quantitative image analysis of the independent test-set images used for four-class phase-state evaluation, including two-phase (n = 352), boundary state (n = 20), single-phase (n = 318), and aggregate-like phenotype (n = 30) groups. Bars represent mean ± s.d.; each data point represents a measurement derived from a test-set image. Pairwise statistical comparisons were performed using two-sided Mann–Whitney U tests. P values were calculated using the asymptotic method with tie and continuity corrections and adjusted for multiple comparisons within each panel using the Holm method. Detailed P values are provided in Table S1. All adjusted P values were < 0.0001.

A. Quantification of condensate count across the four condensate states.

B. Quantification of condensate area across the four condensate states.

C. Quantification of equivalent condensate diameter across the four condensate states.

D. Quantification of the coefficient of variation (CV) across the four condensate states.

E. Quantification of the partition coefficient (K) across the four condensate states.

**Figure S10.** (A-E) Training and validation loss across five folds.

**Figure S11.** Performance comparison of lightweight backbone models. Class 1–4 correspond to two-phase, single-phase, boundary state, and aggregate-like phenotype, respectively.

(A–D) Confusion matrix (Class 1–4 correspond to two-phase, single-phase, boundary state, and aggregate-like phenotype), Training and Validation loss curves, Precision–Recall curves and multi-class ROC curves of ResNet-18.

(E–H) Confusion matrix (Class 1–4 correspond to two-phase, single-phase, boundary state, and aggregate-like phenotype), Training and Validation loss curves, Precision–Recall curves and multi-class ROC curves of EfficientNet-B0.

**Figure S12.** Representative micrographs showing heparin sodium–induced droplet dissolution (C_tot_ = 203.36 µM).

**Figure S13.** Comparison of RBF-SVM–reconstructed 3D phase diagrams generated using experimentally measured phase-state data acquired at 25, 31, 37, 41, and 45°C versus those generated using data acquired only at 25, 37, and 45°C.

A. 3D phase diagram of the Krt1 condensate system with the experimentally measured phase-state data at 31 and 41°C included in the RBF-SVM fitting.

B. 3D phase diagram of the Krt1 condensate system with the experimentally measured phase-state data at 31 and 41°C excluded from the RBF-SVM fitting. For visual comparison, the experimentally measured phase-state labels at 31 and 41°C are displayed with increased transparency but were not used in the RBF-SVM fitting.

C. High-resolution local 3D phase diagram with the experimentally measured phase-state data at 31 and 41°C included in the RBF-SVM fitting.

D. High-resolution local 3D phase diagram with the experimentally measured phase-state data at 31 and 41°C excluded from the RBF-SVM fitting. For visual comparison, the experimentally measured phase-state labels at 31 and 41°C are displayed with increased transparency but were not used in the RBF-SVM fitting.

E. 3D phase diagram of the heparin sodium–modulated system with the experimentally measured phase-state data at 31 and 41°C included in the RBF-SVM fitting.

F. 3D phase diagram of the heparin sodium–modulated system with the experimentally measured phase-state data at 31 and 41°C excluded from the RBF-SVM fitting. For visual comparison, the experimentally measured phase-state labels at 31 and 41°C are displayed with increased transparency but were not used in the RBF-SVM fitting.

G. Exploratory 3D phase diagram over a broad concentration range with the experimentally measured phase-state data at 31 and 41°C included in the RBF-SVM fitting.

H. Exploratory 3D phase diagram over a broad concentration range with the experimentally measured phase-state data at 31 and 41°C excluded from the RBF-SVM fitting. For visual comparison, the experimentally measured phase-state labels at 31 and 41°C are displayed with increased transparency but were not used in the RBF-SVM fitting.

**Figure S14.** Direct comparison between experiment-informed and RBF-SVM–interpolated 2D phase maps at the intermediate temperatures of 31 and 41°C. The experiment-informed 2D phase maps at 31 and 41°C were extracted as temperature-specific slices from the RBF-SVM–reconstructed 3D phase diagrams incorporating experimentally measured phase-state labels acquired at 25, 31, 37, 41, and 45°C. In contrast, the RBF-SVM–interpolated 2D phase maps at 31 and 41°C were obtained from the original RBF-SVM model fitted exclusively to experimentally measured phase-state labels acquired at 25, 37, and 45°C. For direct comparison and validation, the experimentally measured phase-state labels at 31 and 41°C were overlaid on the corresponding interpolated 2D phase maps but were not used in fitting the original RBF-SVM model.

A. Comparison of the experiment-informed and RBF-SVM–interpolated 2D phase maps of the Krt1 condensate system at 31°C.

B. Comparison of the experiment-informed and RBF-SVM–interpolated 2D phase maps of the Krt1 condensate system at 41°C.

C. Comparison of the experiment-informed and RBF-SVM–interpolated high-resolution local 2D phase maps at 31°C.

D. Comparison of the experiment-informed and RBF-SVM–interpolated high-resolution local 2D phase maps at 41°C.

E. Comparison of the experiment-informed and RBF-SVM–interpolated 2D phase maps of the heparin sodium–modulated system at 31°C.

F. Comparison of the experiment-informed and RBF-SVM–interpolated 2D phase maps of the heparin sodium–modulated system at 41°C.

G. Comparison of the experiment-informed and RBF-SVM–interpolated exploratory 2D phase maps over a broad concentration range at 31°C.

H. Comparison of the experiment-informed and RBF-SVM–interpolated exploratory 2D phase maps over a broad concentration range at 41°C.

## Supporting Table:

Table S1. Holm-adjusted P values for pairwise comparisons in Figure S9A–E

| **Comparison** | **A** | **B** | **C** | **D** | **E** |
| --- | --- | --- | --- | --- | --- |
| Two-phase vs Boundary state | 1.762 × 10^-13^ | 5.225 × 10^-10^ | 3.268 × 10^-10^ | 5.242 × 10^-7^ | 2.732 × 10^-12^ |
| Two-phase vs Single-phase | 4.204 × 10^-123^ | 4.202 × 10^-123^ | N/A | N/A | N/A |
| Two-phase vs Aggregates | 7.073 × 10^-5^ | 2.964 × 10^-18^ | 2.515 × 10^-17^ | 2.956 × 10^-19^ | 2.434 × 10^-17^ |
| Boundary state vs Single-phase | 1.493 × 10^-74^ | 1.493 × 10^-74^ | N/A | N/A | N/A |
| Boundary state vs Aggregates | 4.426 × 10^-8^ | 9.870 × 10^-9^ | 9.830 × 10^-9^ | 6.022× 10^-9^ | 2.954 × 10^-9^ |
| Single-phase vs Aggregates | 1.582 × 10^-76^ | 1.582 × 10^-76^ | N/A | N/A | N/A |

**Note:** Reported *P* values are Holm-adjusted values based on two-sided Mann–Whitney U tests using the asymptotic method with corrections for ties and continuity. The Holm adjustment was performed separately within each panel using only the valid pairwise comparisons. The complete test set comprised n = 352 two-phase images, n = 20 boundary-state images, n = 318 single-phase images, and n = 30 aggregate-like images. For Figure S9C–E, the corresponding metrics were undefined for single-phase images because no condensates were detected. The single-phase group was therefore excluded from these statistical analyses, and comparisons involving this group are indicated as not applicable (N/A). All calculated Holm-adjusted *P* values were < 0.0001.

Table S2. Comparison of phase-diagram mapping platforms

| **Platform** | **Key technical features** | **Phase diagram** | **Throughput** | **Temperature control** | **Sample consumption** | **End-to-end time** | **Phase-state classification** |
| --- | --- | --- | --- | --- | --- | --- | --- |
| **Centrifugal microfluidic chip**  Li et al. [27] | Centrifugal concentration gradient + natural evaporation-induced concentration. | 2D  phase diagram | 25 reaction units;  experimental: >200 concentrations | 37 °C constant-temperature | Not reported | 150 min | Manual image-based readout |
| **Label-free droplet microfluidics** Villois et al. [28] | Droplet microfluidics + label-free brightfield imaging | 2D phase diagram | Not reported | 25-55 °C | Several hundred microliters | Not reported | Brightfield image analysis |
| **PhaseScan**  Arter et al. [29] | Droplet microfluidics + fluorescence barcoding + SVM classification | 2D/3D phase diagrams | 2600 droplets/experiment | Not applicable | 10-20 µL | Not reported | SVM-based phase-state classification |
| **PhaseXplorer**  Jansen et al. [30] | Droplet microfluidics + CNN recognition + active learning | 3D/4D phase diagrams | >1000 droplets/sample | Not applicable | Not reported | ≥70 min;  (7min/iteration; ≥10 iterations) | CNN-based image recognition and active-learning-guided classification |
| **T-PhaseMap**  This work | Centrifugal concentration gradient + temperature control + deep-learning four-class classification | 3D phase diagram | 54 reaction units;  18 concentrations × 3 temperatures | 25, 31, 37, 41,45°C  (adjustable) | 180 µL/chip | <30 min | Deep-learning-based four-class phase-state classification |

Table S3. Quantitative validation of binary LLPS/non-LLPS classification by the original RBF-SVM model at 31 and 41°C across four phase-diagram datasets

| **Phase diagram** | **Accuracy (%)** | **Correctly classified points (n/N)** | **Misclassified points (n/N)** | **Misclassified LLPS  points (n/N)** | **Misclassified non-LLPS points (n/N)** |
| --- | --- | --- | --- | --- | --- |
| Krt1 condensate phase diagram | 95.83 | 69 / 72 | 3 / 72 | 1 / 37 | 2 / 35 |
| High-resolution local phase diagram | 94.44 | 68 / 72 | 4 / 72 | 3 / 44 | 1 / 28 |
| Heparin sodium-modulated phase diagram | 97.22 | 70 / 72 | 2 / 72 | 1 / 37 | 1 / 35 |
| Exploratory phase diagram | 98.61 | 71 / 72 | 1 / 72 | 1 / 46 | 0 / 26 |

**Note:** Values are reported as n/N. For this binary validation, points classified as two-phase, boundary-state, or aggregate-like phenotypes were assigned to the LLPS class, whereas points classified as single-phase states were assigned to the non-LLPS class. This grouping follows the binary LLPS/non-LLPS labeling scheme used to define and reconstruct the RBF-SVM phase boundary. For each phase diagram, 72 intermediate-temperature points were evaluated in total, comprising 36 points at 31°C and 36 points at 41°C. In n/N, n denotes the number of points indicated by the corresponding column, and N denotes the relevant denominator. Accuracy = correctly classified points / total points × 100%.
